# Ultrasound versus traditional formula for subglottic diameter-based uncuffed endotracheal tube selection in pediatric patients

**DOI:** 10.6026/973206300222338

**Published:** 2026-04-30

**Authors:** Urmila Keshari, Rajni Thakur, Sherin Soni, Kaushal R.P, Lovely Kaushal, Ajay Kumar Yadav

**Affiliations:** 1 Department of Anaesthesiology, Gandhi medical College, Bhopal, India; 2 Department of Radiology, Gandhi medical College, Bhopal, India; 3 Consultant anaesthesiologist, Aster Prime Hospital, Hyderabad, India

**Keywords:** Pediatric airway, ultrasonography, endotracheal tube (ETT), subglottic diameter, Cole's formula

## Abstract

Accurate endotracheal tube (ETT) size selection is vital in pediatric anesthesia, yet traditional age-based formulas such as the modified Cole's formula are often unreliable; ultrasonography (USG), by directly visualizing the subglottic airway, offers a more precise alternative. In a cross-sectional study of 75 children aged 1-8 years, elective cases underwent ultrasound measurement of the mean transverse subglottic diameter, while emergency cases relied on the age-based formula. Ultrasound demonstrated superior accuracy, reducing ETT changes (5% vs. 20%), second intubation attempts (5% vs. 20%) and post-intubation complications (4.5% vs. 16.5%). It also showed higher sensitivity (100% vs. 96%) for predicting appropriate tube size. Thus, we show ultrasonography as a more reliable method than traditional formulas and recommend its integration into routine pediatric airway assessment.

## Background:

Accurate selection of endotracheal tube (ETT) size in pediatric anesthesia is essential to minimize airway resistance, prevent mucosal trauma and avoid complications such as postoperative sore throat, glottic edema and subglottic stenosis [3]. The pediatric airway differs anatomically from adults, with the narrowest segment being the subglottic region at the cricoid level [7]. Because small deviations in tube diameter can significantly affect airway safety, appropriate sizing is critical. Age based formulas particularly the modified Cole's formula has been used for decades due to ease of application. However, their accuracy varies widely and is often suboptimal. Studies report that age-based formulas select the correct ETT size in only 31-60% of cases [2, 3]. These formulas estimate size based on external physical parameters rather than directly measuring airway dimensions. Ultrasonography provides a noninvasive, real-time assessment of subglottic diameter and has shown strong correlations with optimal ETT size in multiple pediatric studies [1, 4 and 5]. Therefore, it is of interest to evaluate whether ultrasound measurement of subglottic diameter enhances accuracy in uncuffed ETT size selection compared to the traditional age-based formula.

## Materials and Methods:

This observational cross-sectional study was conducted at the Department of Anaesthesiology, Gandhi Medical College and associated hospitals, Bhopal. The study included 75 children aged 1-8 years (ASA I-II) scheduled for surgery under general anesthesia. Ethical approval and parental consent were obtained.

## Ultrasonography protocol:

Elective patients underwent ultrasonography during pre-anesthetic evaluation. A 7-12 MHz linear probe was applied in the midline with the neck in the sniffing position. The radiologist identified the vocal cords and moved caudally to visualize the cricoid arch. The minimal transverse subglottic air-column diameter was measured, corresponding to the narrowest region of the pediatric airway. Ultrasound has been validated in prior studies, showing high correlation between ultrasonographic measurements and ETT outer diameter [1, 2 and 4].

## ETT size prediction (Table 1 - see PDF):

[1] Elective cases: Both ultrasound prediction and Cole's formula were used.

[2] Emergency cases: Only Cole's formula was used due to time constraints.

Intubation was performed by senior anesthesiologists using standard anesthesia techniques. Proper tube placement was confirmed by capnography and bilateral auscultation. An air-leak test determined the adequacy of tube size.

## Outcome measures:

[1] Accuracy of predicted ETT size

[2] Rate of ETT size change

[3] Number of intubation attempts

[4] Sensitivity of prediction methods

[5] Post-intubation complications (sore throat, cough, dysphonia, stridor)

## Statistical analysis:

Chi-square test, Pearson correlation coefficients and descriptive statistics were used. P < 0.05 was considered significant.

## Results and Discussion:

This study demonstrates that ultrasonography is significantly more accurate than the modified Cole's formula in predicting uncuffed ETT size. These results align strongly with previous research. Ultrasonographic measurement of the subglottic diameter shows robust correlation with optimal ETT size (Table 3, Figure 2 - see PDF). Shibasaki *et al.* found a strong correlation between ultrasound- measured airway diameters and correct ETT sizing [1]. Bae *et al.* showed ultrasound correctly predicted ETT size in 60% of children, compared to 31% using age-based formulas [2]. Bhut *et al.* and recent study in similar populations [11] reported that ultrasound outperformed traditional formulas in clinical prediction [3]. The present study's results-ETT change rates of 5% with USG vs. 20% with formula-support those earlier findings. Likewise, a higher first-attempt success rate (95% vs. 80%) demonstrates the clinical advantage of ultrasound (Table 2, Figure 1 - see PDF). Prior studies have shown similar improvements in intubation success with ultrasound-guided prediction [4, 6]. Post- intubation complications were markedly lower with ultrasound (Table 4, Figure 3 - see PDF). Complications such as dysphonia and stridor (seen only with formula-based sizing) may reflect mucosal trauma from oversized tubes, consistent with observations from Kim *et al.* and Altun *et al.* [5, 6]. Massive reductions in total complications (4.5% vs. 16.5%) reinforce ultrasound's superiority. These findings echo results in several contemporary pediatric airway studies [7, 8 and 9]. Ultrasound also demonstrated perfect sensitivity (100%), outperforming the formula. Comparable sensitivity advantages have been shown in systematic reviews evaluating pediatric airway ultrasound [10]. Collectively, this evidence suggests that USG is a more reliable, individualized and safer method for ETT size prediction compared to traditional formulas.

## Conclusion:

Ultrasonography is a highly accurate and reliable method for selecting uncuffed endotracheal tube size in pediatric patients. Compared to the age-based Cole's formula, it significantly reduces ETT changes, increases first-attempt success and decreases post-intubation complications. USG should be integrated into routine pediatric airway assessment whenever feasible.

## Future recommendations:

[1] Routine use of airway ultrasonography in elective pediatric surgeries.

[2] Development of standardized, age-stratified ultrasound-based predictive formulas.

[3] Training of anesthesia residents in pediatric airway ultrasonography.

[4] Evaluation of portable ultrasound devices for emergency settings.

[5] Larger multicenter trials to validate effectiveness across diverse populations.

[6] Assessment of USG utility in neonates and children with airway anomalies.
